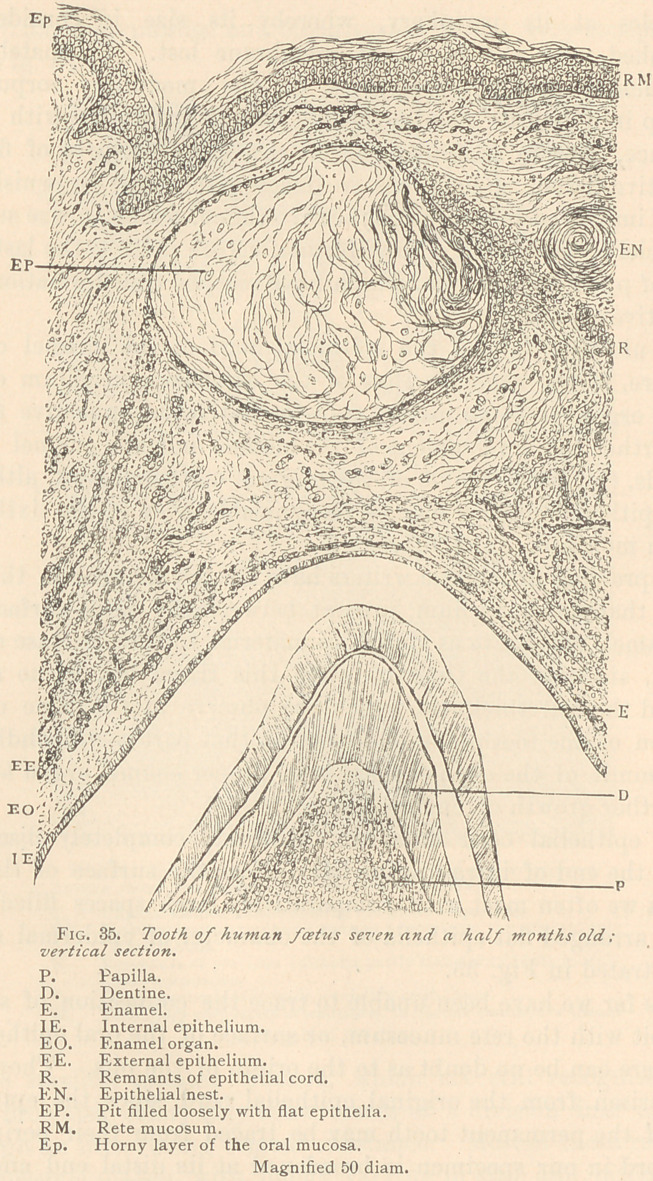# Contributions to the History of Development of the Teeth

**Published:** 1887-10

**Authors:** Carl Heitzmann, C. F. W. Bödecker


### Independent Practitioner.

Vol. VIII. October, 1887. No. 10.

Note.—No paper published or to be published in another journal will be accepted for this department. All papers must be in the hands of the Editor before the first day of the month preceding that in which they are expected to appear. Extra copies will be furnished to each contributor of an accepted original article, and reprints, in pamphlet form, may be had at the cost of the paper, press-work and binding, if ordered when the manuscript is forwarded. The Editor and Publishers are not responsible for the opinions expressed by contributors. The journal is issued promptly. on the first day of each month.

### Original Communications.

### CONTRIBUTIONS TO THE HISTORY OF DEVELOPMENT OF THE TEETH.

BY CARL HEITZMANN, M. D., AND C. F. W. BÖDECKER, D. D. S., M. D. S.

Continued From Page 462.

### IV. THE EPITHELIAL CORD OF THE ENAMEL ORGAN.

The next question to be considered is, how does epithelium grow from an originally small point into a comparatively long epithelial cord? Most observers agree that epithelium has an independent growth, and that its elements by division and multiplication will produce epithelium, and no other tissue. During the last fourteen years many microscopists have studied the so-called indirect division of "individual cells," which became traceable after the application of certain reagents, especially saffranine. This dye rendered visible a filamentous structure in the nucleus, apparently independent of the surrounding protoplasm, which did not take up the stain of the saffranine. The filaments produced beautiful star-shaped figures, with equatorial divisions leading to a fission of the original nucleus, and the process has been termed *karyokinesis* or *mitosis*.

In fresh specimens, or those preserved in a chromic acid solution, the filamentous structure of the nucleus does not exist, or, at least, is not plainly visible, although Bizzozero, of Italy, recently claimed that saffranine will bring out the filamentous structure of the nucleus even in chromic acid specimens. The filaments taking up the dye were termed chromatine, and all those remaining pale, achromatine; and it was claimed that these were two different sub-

FIG. 32. *Epithelial cord of the enamel organ of a human embryo three and a half months old.*

E. Epithelial cord.

C. Connective tissue.

Magnified 800 diam.

stances. Considering the fact that the filaments are produced only by certain reagents, we should be loath in assuming that the structure thus rendered visible really corresponds to the unstained and living epithelium. The doubts become still stronger if we recall the fact that living matter is stained deeply by the same reagent, if present in a compact mass, where thin layers of it remain unstained, as is the case in the protoplasm which surrounds the central nucleus. From this point of view the terms chromatine and achromatine become superfluous, especially if we admit that the living matter during life is in constant motion, particularly in the process of growth, although its continuity may temporarily be interrupted.

What we see in a growing epithelial cord, with high powers of the microscope, is depicted in Fig. 32.

We observe epithelia along the borders of the cord which are elongated and bear the name of columnar epithelium, while the central portion is filled with epithelia, exhibiting about an equal diameter in all directions, and which are termed cuboidal. Both varieties show differences in the size and structure of their nuclei. Some nuclei are very large and distinctly reticular, others are small and nearly compact, often appearing as if split up into several lumps, with a vacuole or plasmatic space. Sometimes a nucleus is elongated or irregular in shape, another being globular or vesicular. Again, we see epithelia much enlarged, holding in their interior several nuclei, a condition which has been termed by previous observers "the mother cell." Not infrequently we observe solid, spindle-shaped bodies in the cement substance, between two epithelia. All these forms become intelligible only under the assumption that epithelium is composed of protoplasm, in which the living matter greatly varies in size and shape, according to the state of growth and new formation. The cement substance is often absent, and thus large protoplasmic masses become conspicuous, with a varying number of nuclei. Where the cement substance is present, it is usually traversed with radiating lines, which are the connecting bridges of the living matter. Again, these lines may coalesce into solid masses, presenting spindle, pear, or club-shapes, from which new epithelia arise, as shown by Louis Elsberg.

The way, therefore, in which epithelium grows, is by augmentation of its living matter, and the appearance of new cement substance—that is, new lines of demarkation, in which process the continuity of the living matter—though temporarily interrupted in certain foci—in the whole remains preserved and unbroken. In the epithelial cord of the enamel organ the connecting spokes in the cement substance are prominently marked in all its layers and stages of development.

Of special interest are concentrically stratified globular nests and buds, in which the epithelia appear flattened and arranged in the shape of an onion. Such nests are often lacking altogether, and sometimes they are present in small numbers in the center or at the periphery of the epithelial cord. Sometimes their number is very large, as represented in Fig. 1 (page 227). The centers of the nests are occasionally filled with globules of high refraction, possibly colloid material or elaidine (horn fat). In some specimens the epithelial structure of the peg is little marked, especially in places where the epithelial peg produces broadened layers, without sharp contours toward the surrounding connective tissue. In such places tracts are seen composed of rows of solid nuclei, or solid cords, between which fine granular protoplasm is visible. Such tracts have been repeatedly alluded to in the description of the early forms of development of the epithelial cord of the enamel organ. It is quite possible that in such places a transition takes place from the epithelial to the medullary, and from this to connective tissue. This is rendered probable by the fact that the broadened portions of the epithelial cord have sharp contours only on one side, whereas the opposite periphery almost blends with the adjacent connective tissue, without a distinct line of demarkation between the two kinds. While we admit that the original epithelial peg and cord is of a marked epithelial structure, at the same time we claim that in the advancing process of growth the epithelium does not retain its specific structure, but blends with or is transformed into connective tissue.

Let us now ask the question: What is the ultimate fate of the epithelial cord of the enamel organ?

In the earliest stages of its development we meet with numerous lateral offshoots and sprouts, which, preceding their ultimate disappearance, are visible in the shape of medullary corpuscles. The destiny of the cord is obviously the production of the enamel organ for the benefit of the formation of the enamel. This process is accomplished with the fifth month of intra-uterine life, with which the writers began the description of the formation of the enamel. In the latter part of the fourth and in the fifth month, the original epithelial cord undergoes peculiar changes, which have attracted the attention of many previous observers. The main change consists in the breaking up of the epithelial cord into innumerable clusters of a more or less marked epithelial structure, between which fibrous connective tissue has appeared, completely isolating the clusters. Changes of this description are best seen in horizontal sections of the jaws. (See Fig. 33.)

With low powers of the microscope, we observe a large number of clusters which are distinctly marked in specimens preserved in chromic acid solution. They have a brownish color, and are either sharply contoured or more or less blending with the adjacent connective tissue. Many of these clusters hold concentrically arranged epithelial nests, and, judging from their size and shape, they must have originated from a very active new growth of epithelium, which is indicated also by a number of buds sprouting from the original clusters. The buds have been pushed apart by fibrous connective tissue, since many of them appear entirely isolated, and as if imbedded in the fibrous connective tissue. Higher powers of the microscope reveal the fact that the process of dissolution of the original epithelial cord is identical with that of the external epithelium of the enamel organ, the only difference being that in the latter process numerous newly formed blood-vessels participate, whereas, in the breaking up of the epithelial cord, the new formation of blood-corpuscles and blood-vessels is not very conspicuous. (See Fig. 34.)

The smallest isolated clusters, which are still recognizable by their brownish color, only show traces of a division into epithelia through an intervening cement substance. Most of them represent protoplasmic masses, with nuclei varying in size, and interspersed at more or less regular intervals. Such clusters are often found surrounded by an almost homogeneous layer of a so-called basement membrane. In the next stage, the cluster splits up into medullary corpuscles at its periphery, whereby its size is considerably diminished, and the basement membrane lost. Ultimately the multinuclear protoplasmic mass, or the medullary corpuscles, split up into delicate spindles, which become infiltrated with basis-substance, thereby assuming the characteristic features of fibrous connective tissue. Sometimes we meet with single brownish corpuscles imbedded in fibrous connective tissue, which in size surpass the nuclei of the latter. Such formations are possibly the last remnants of previous epithelia, which have escaped transformation into connective tissue.

The ultimate fate of the epithelial cord of the enamel organ, therefore, is the same as that of the external epithelium of the enamel organ, it being partly transformed into connective tissue. The further the development of the enamel and the enamel organ proceeds, the less is visible of the original epithelial cord, although small epithelial clusters may be recognizable even in the sixth and seventh month of fœtal life.

In a previous article the writers have already alluded to the possibility that the epithelium, present between the outer surface and the enamel, may serve as stored-up material for the increase of the enamel, since at the time of birth this tissue has by no means attained the full thickness which we observe at the time of the eruption of the tooth, and also since on that part corresponding to the summit of the enamel there is no proper enamel organ left for the further growth of enamel tissue.

The epithelial cord does not, however, completely disappear toward the end of intra-uterine life. Near the surface of the oral mucosa we often meet with comparatively large spaces filled with loosely arranged flat epithelia of the character of epidermal scales, as illustrated in Fig. 35.

Thus far we have been unable to trace the connection of such a large pit with the rete mucosum, or surface of the oral epithelium. But there can be no doubt as to the origin of the pits. They must have arisen from the original epithelial cord, since the epithelial cord of the permanent tooth may be traced from their periphery. The cord in our specimen is broadened at its distal end and surrounded by a small papilla corresponding in every respect to the developing temporary tooth in an embryo of about three and a half months. The epithelia filling the pit are arranged loosely, simi-

FIG. 35. *Tooth of human fœtus seven and a half months old; vertical section.*

P. Papilla.

D. Dentine.

E. Enamel.

IE. Internal epithelium.

EO. Enamel organ.

EE. External epithelium.

R. Remnants of epithelial cord.

EN. Epithelial nest.

EP. Pit filled loosely with flat epithelia.

RM. Rete mucosum.

Ep. Horny layer of the oral mucosa.

Magnified 50 diam.

lar to those of the horny layer of the oral mucosa, and many of them hold glistening granules of what possibly is elaidine. The boundary of the pit is made up of a single row of cuboidal epithelia, which in some places may produce stratified hills and protrusions. The pit corresponds to the summit of the temporary tooth, and its destiny seems to be to prearrange the route for its eruption. Between the lower periphery of the pit and the upper boundary of the enamel organ, or rather its external epithelium, the connective tissue is loose and approaches the myxomatous structure, containing very small groups of epithelium or medullary corpuscles sprung therefrom. Such clusters are especially conspicuous in the space between the epithelial cord of the permanent tooth and the external epithelium of the enamel organ, where a large number of capillary blood vessels is also visible.

From these investigations the writers derived the following corollaries:

I. The epithelial cord of the enamel organ is a formation of the epiblast.

II. In the same manner as the nerve centers (brain and spinal cord) are products of the epiblast, greatly changing their character in the further course of development, the epithelial cord gives rise to the myxomatous tissue of the enamel organ.

III. The epithelial cord arises from a furrow lined with epithelium, about the sixth week of intra-uterine life, and grows obliquely downward into the connective tissue, which latter produces the papilla about the third fœtal month.

IV. After the formation of the enamel organ the epithelial cord is dissolved into clusters which are partly retained in, and partly transformed into, fibrous connective tissue.

V. The remnants of the external epithelium, as well as those of the epithelial cord, very probably furnish the material for the increase of the enamel after the original enamel organ has been exhausted.

VI. The epithelial cord of the temporary tooth furnishes a lateral offshoot for the formation of the permanent tooth. The papillæ of the latter appear about the seventh month of intra-uterine life.

VII. From the epithelial cord, corresponding to the summit of the temporary tooth, spaces are formed which are filled with flat epithelia. Such spaces serve probably as guides for the eruption of the tooth.

(TO BE CONTINUED.)

## Figures and Tables

**Fig. 32. f1:**
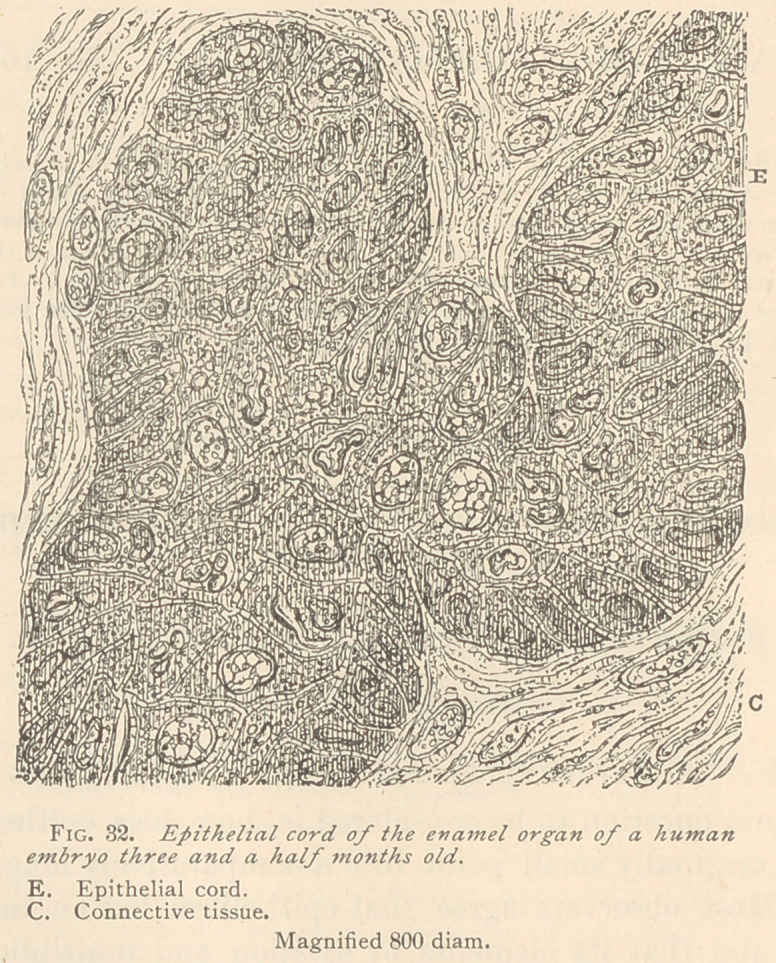


**Fig. 33. f2:**
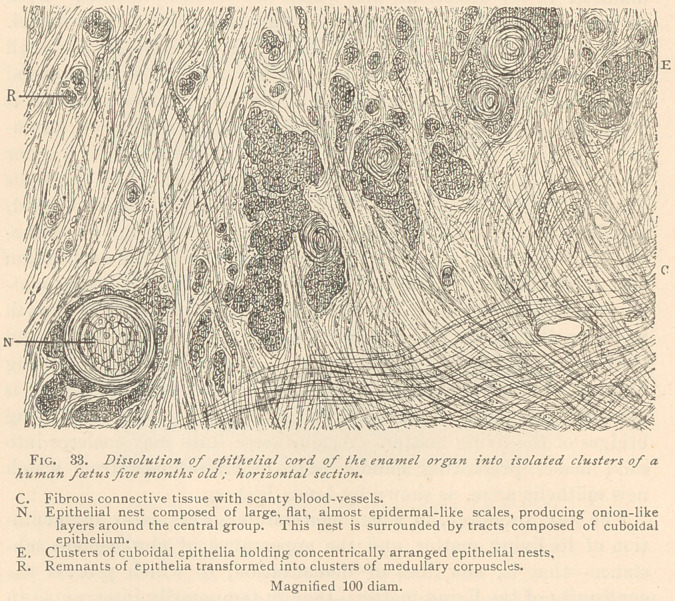


**Fig. 34. f3:**
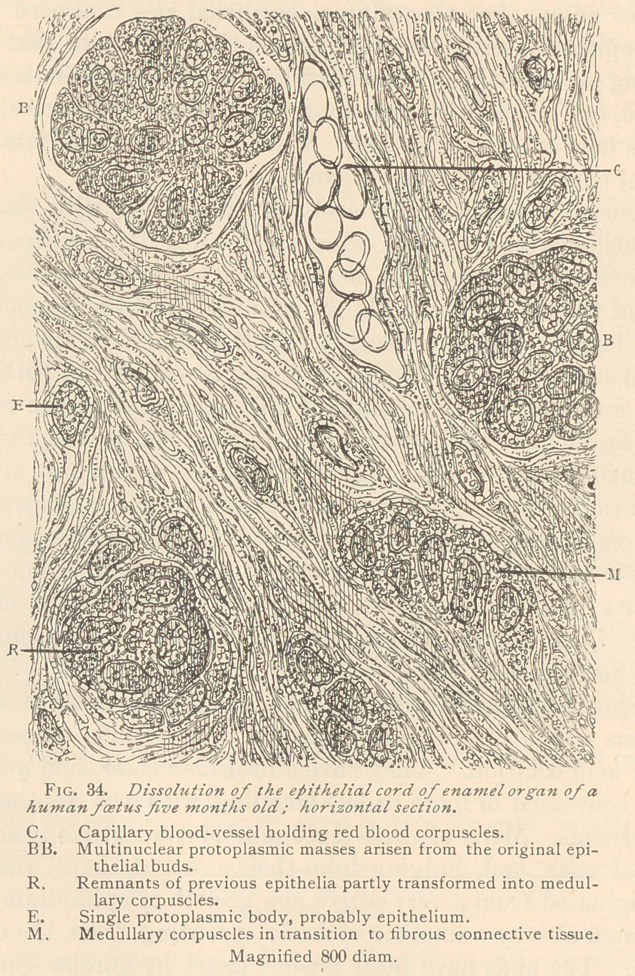


**Fig. 35. f4:**